# Technical note: The effect of the 4‐mm‐collimator output factor on gamma knife dose distributions

**DOI:** 10.1120/jacmp.v4i4.2512

**Published:** 2003-09-01

**Authors:** John P. Gibbons, Dimitris Mihailidis, Curtis Worthington, Hassaan Alkhatib, Raleigh Boulware, Robert Clark, Burke Dial, William Neglia

**Affiliations:** ^1^ Gamma Knife Center of the Carolinas Columbia South Carolina 29203

**Keywords:** collimator factor, helmet factor, gamma knife, radiosurgery

## Abstract

We present results of investigations of the clinical significance of variations in the value of the 4‐mm‐collimator output factor, ${\text{OF}}_{4/18}$. Changes in treatment volume, dose‐volume histograms (DVHs), and isodose distributions were studied, by varying ${\text{OF}}_{4/18}$ up to 20%. The variations were performed on a sample of clinical patient treatment plans for which the 4 mm collimator was used. Although smaller effects are noted for the prescription isodose line, greater dosimetric changes occur for higher dose regions within the target.

PACS number(s): 87.53.–j, 87.52.–g

The Gamma Knife collimator output factor (or helmet factor) is defined as the dose rate delivered by a particular helmet divided by the dose rate delivered by the reference (18‐mm) collimator helmet. The experimental determination of the 4‐mm‐collimator output factor, ${\text{OF}}_{4/18}$, is difficult, because of the small size of the radiation field relative to the sizes of traditional radiation dosimeters. A number of experimental techniques have been proposed^1^ ^‐^ ^6^ for the measurement of this factor in an effort to improve the accuracy of delivered dose. Because of the difficulties involved in this measurement, these studies have reported large variations in ${\text{OF}}_{4/18}$, with values ranging from 0.63 to 0.92.

In 1998, the manufacturer, Elekta, Inc., recommended^7^ changing the factor by almost 10% (i.e., 0.80–0.87), based on a compilation of measurements made at four Gamma Knife units in Europe. In many centers, this recommendation has created a dilemma as to whether to incorporate the new factor in light of previous clinical experience. Elekta recommended an adjustment of prescription for cases where the 4 mm collimator is used alone, when the dose level prescribed has already been empirically established.^7^ For treatment plans where 4 mm collimators are used with those of other sizes, no significant changes were anticipated. The purpose of this study was to make a more quantitative assessment of changes in ${\text{OF}}_{4/18}$ for plans with multiple collimators.

Changes in isodose distributions, dose‐volume histograms (DVHs) and treatment volumes due to variations in the 4‐mm‐helmet factor have been investigated using the Gamma Knife treatment planning system, Leksell GammaPlan version 5.20 (LGP). The variations have been performed on a sample of patient clinical treatment plans that incorporate the 4 mm helmet. Spatial resolution was optimized by setting the calculation dose grid to the smallest value that still encompassed the prescription isodose surface. The dose grid ranged from 0.4 to 1.9 mm for the plans investigated in this work. Treatment volume and DVHs have been determined using the measurement tools available on the planning system.

In all treatment plans in this study, a value of ${\text{OF}}_{4/18}=0.870$ was used in LGP. If the true value of ${\text{OF}}_{4/18}$ is smaller or larger, the dose delivered from each 4 mm shot will be proportionally smaller or larger than that predicted by the treatment plan. Dosimetric changes associated with these differences were investigated by varying the weights of each 4 mm shot within the plan by a fixed percentage. The weights assigned to other collimator shots were kept constant. In almost all cases, variation of the 4‐mm‐shot weights by this magnitude influenced the dose at the reference point, taken as the maximum dose within the matrix. In these cases, the reference dose is adjusted so that the treatment times for the other shots remained constant.

Prior to changing collimator weights, a new region of interest was created, entitled “50% IDL.” This region was contoured by tracing the prescription isodose line on each axial MR image. Changes in the dose distribution were then observed by comparing this region with the new prescription isodose line obtained with different 4‐mm‐collimator weighting.

Treatment volumes were compared using the histogram feature within the measurement toolbar. A histogram of the calculation matrix was generated and the volume of the prescription isodose was measured for each weight variation. In cases where the reference dose has been adjusted, the prescription isodose is taken as the dose (in Gy, not percent) that was intended in the original plan. This may not correspond to the same isodose percentage if the maximum dose is altered.

DVHs were also compared for a sample of patient plans. In these cases, a histogram was generated of the outlined target volume. These histograms were compared for both the original and weight‐altered plans.

Displayed in Fig. 1 is an example of a patient plan used in this study. Shown are sequential 1‐mm‐axial MR images for an acoustic neuroma patient treated at our facility. In this example, 14 shots (eight 8‐mm and six 4‐mm shots) were used to deliver 13 Gy to the 50% isodose surface. On many slices, two contour lines are visible in this figure. The outer (purple) line represents the region “50% IDL,” which was contoured using the current Elekta‐recommended value for the 4‐mm‐helmet output factor: ${\text{OF}}_{4/18}=0.870$. The inner (yellow) line represents the 50% isodose line obtained when the 4 mm weights were reduced by 20%. This latter case represents the delivered dose distribution when the true ${\text{OF}}_{4/18}$ is 20% less (i.e., 0.696) than that programmed into the planning system. As is evident from the figure, relatively large changes in ${\text{OF}}_{4/18}$ result in only minimal changes to the isodose line corresponding to the prescribed dose. Using the distance measurement tools available in LGP, the absolute distance between isodose lines was measured to determine the maximum deviation. For cases where the 4 mm collimator is used alone, no deviation greater than 1 mm was found in the 50% isodose line for changes in ${\text{OF}}_{4/18}$ of up to 20%. For changes in ${\text{OF}}_{4/18}$ of 10%, no deviations greater than 1 mm were observed for all cases outlined in this study.

**Figure 1:**
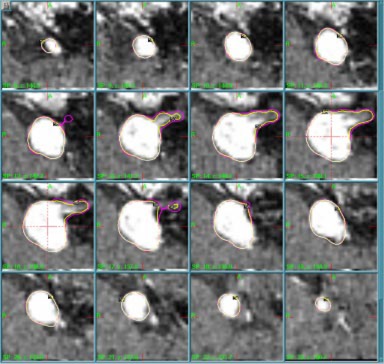
(Color) Treatment plan for an acoustic neuroma patient. Displayed are 16 sequential 1‐mm‐axial MR images through the enhancing target. Displayed are the resulting 50% isodose levels obtained if ${\text{OF}}_{4/18}$ is the same (purple) or 20% smaller (yellow) than that input into the treatment planning system (${\text{OF}}_{4/18}=0.870$).

**Table I:** Changes in treatment volume as a result of variations in the 4‐mm‐collimator output factor. Changes are displayed for patients with metastases (MT), acoustic neuromas (AC), astrocytomas (AS), arteriovenous malformations (AVM), and meningiomas (MN).

						Adjusted volume (cc)		
Patient No.	Disease	No. all shots	No. 4 mm shots	Max dose (Gy)	Volume (cc)	${\text{OF}}_{4/18}$ +10%	${\text{OF}}_{4/18}$ −10%	${\text{OF}}_{4/18}$ +20%	${\text{OF}}_{4/18}$ −20%
1	MT	5	2	34	4.3	4.3	4.3	4.3	4.4
2	AS	17	2	34	10.9	10.9	10.8	10.9	10.8
3	AS	12	2	28	14.9	14.9	14.9	14.9	14.9
4	AS	10	6	29	6.4	6.6	6.3	6.6	6.1
5	MN	9	1	32	8.8	8.9	8.8	8.9	8.8
6	AS	12	2	26	5.8	5.8	5.7	5.8	5.7
7	AVM	4	2	36	0.5	0.6	0.5	0.6	0.4
8	MT	3	1	24	8.8	8.8	8.8	8.8	8.8
9	MT	4	1	44	0.4	0.4	0.4	0.4	0.4
10	AVM	7	2	28	1.6	1.7	1.6	1.7	1.6
11	AS	4	2	28	0.7	0.7	0.8	0.8	0.7
12	AC	14	6	26	2.6	2.6	2.5	2.7	2.4
13	AC	13	4	28	2.2	2.2	2.2	2.3	2.2
14	MN	11	4	28	6.8	6.8	6.8	6.9	6.6
15	AVM	6	2	40	1.4	1.4	1.4	1.4	1.4
16	AC	3	2	28	0.6	0.6	0.6	0.6	0.5
17	MN	4	1	32	1.0	1.0	1.0	1.0	0.9

Additional quantitative information is obtained by viewing dose‐volume histogram data for each case. Displayed in Fig. 2 is a sample of integral dose‐volume histograms for the variations in ${\text{OF}}_{4/18}$ of 10% and 20%. As described above, the reference doses are renormalized such that changes in the 4‐mm‐helmet factor only change 4‐mm‐treatment times. The data indicate that the deviations near the prescription dose level of the treatment isodose are minimal. This level is indicated by the vertical dashed line in Fig. 2. However, the distribution of greater doses within the target is more strongly affected. For example, the volumes which receive at least 18 Gy range from 34% to 43%, depending on the value of ${\text{OF}}_{4/18}$. This effect may be indicative of a steeper dose gradient in the region at or near the prescription isodose. Although the clinical significance of this is unknown, it is evident that a comparison of prescription isodose lines alone may not suffice in evaluating the dosimetric effects.

**Figure 2:**
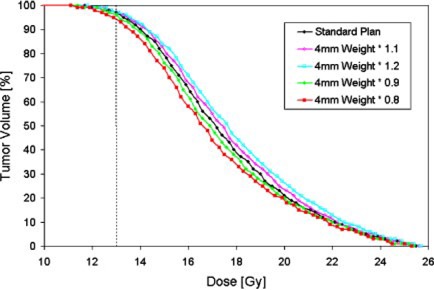
(Color) Dose‐volume histograms for the acoustic neuroma patient displayed in The integral DVHs were obtained with ${\text{OF}}_{4/18}$ variations of ±10% and ±20%. The dashed line indicates the prescription dose value for this patient.

Displayed in Table I are the changes in treatment volumes for a sample of patients treated at our institution. Shown are a subset of investigated patients, each of which received at least one 4 mm shot and at least one larger shot. The table demonstrates the change in treatment volume in cubic centimeters when ${\text{OF}}_{4/18}$ is varied. The increase or decrease in treatment volume is seen to vary only slightly for 10% variations in ${\text{OF}}_{4/18}$. The significance of this variation is also seen to increase with the relative fraction of the 4 mm shots to the total treatment plan.

These data confirm those anticipated by Elekta, which demonstrate little change in treatment volume for large changes in ${\text{OF}}_{4/18}$. Again, however, these data represent the volume determined by that part of the target contained within the prescription isodose. It is likely that the changes in higher dose volumes will be more significant.
